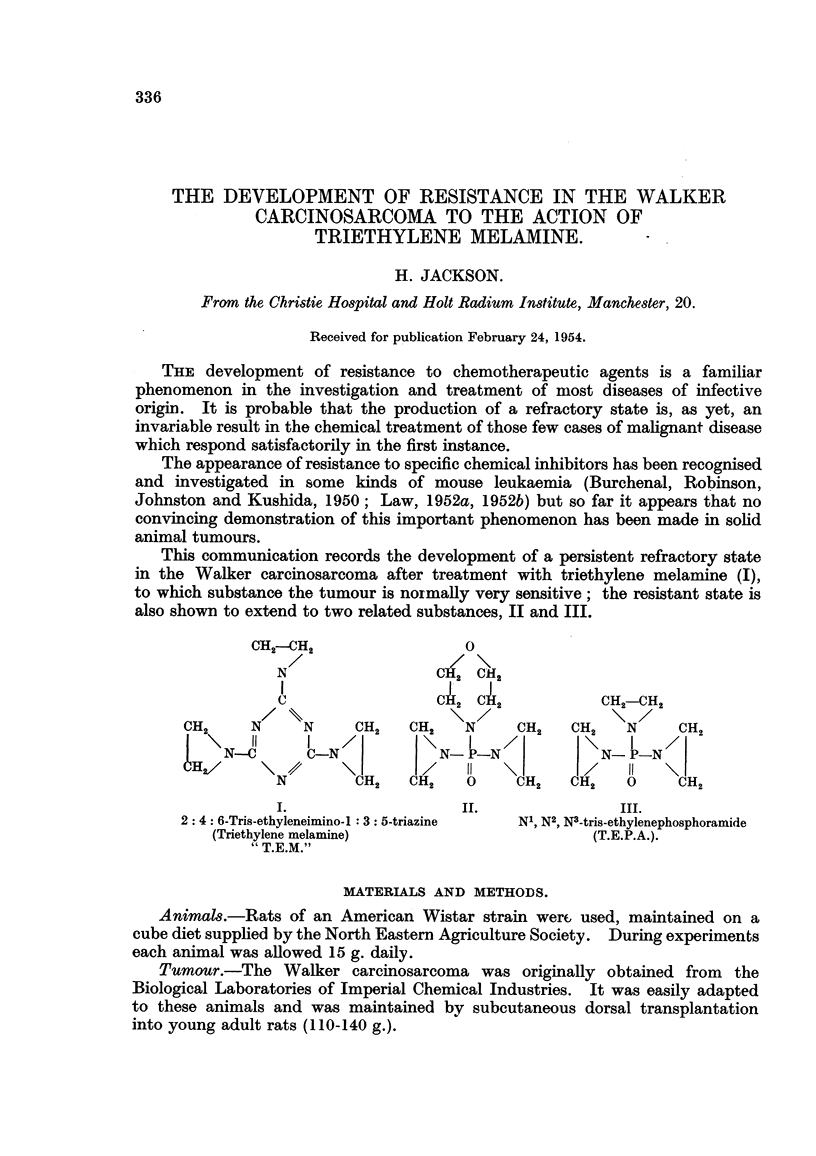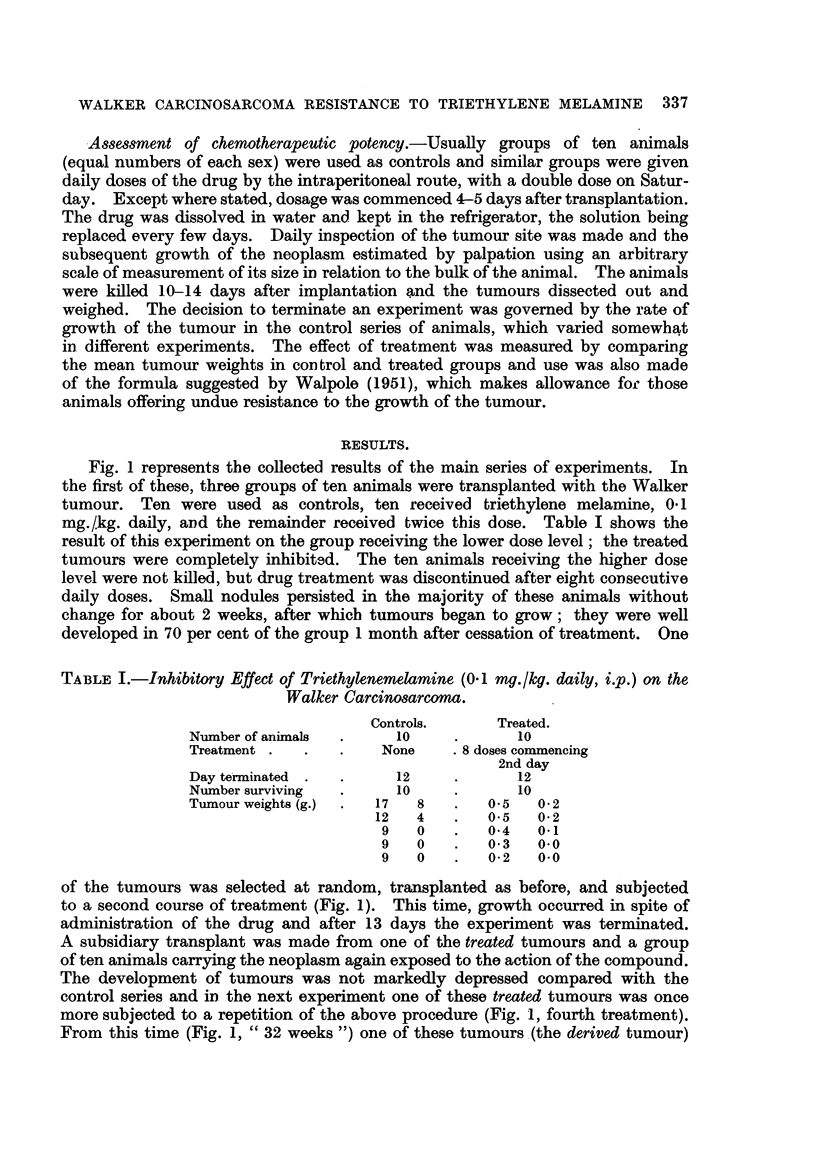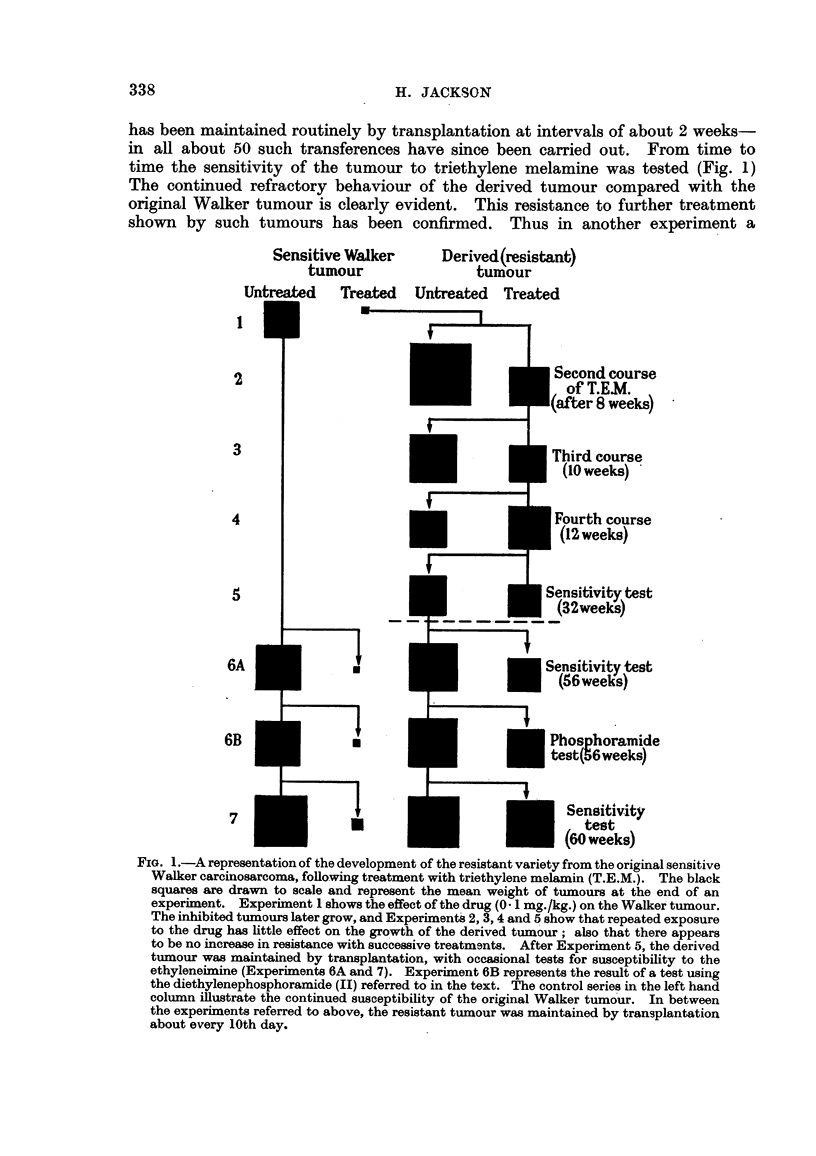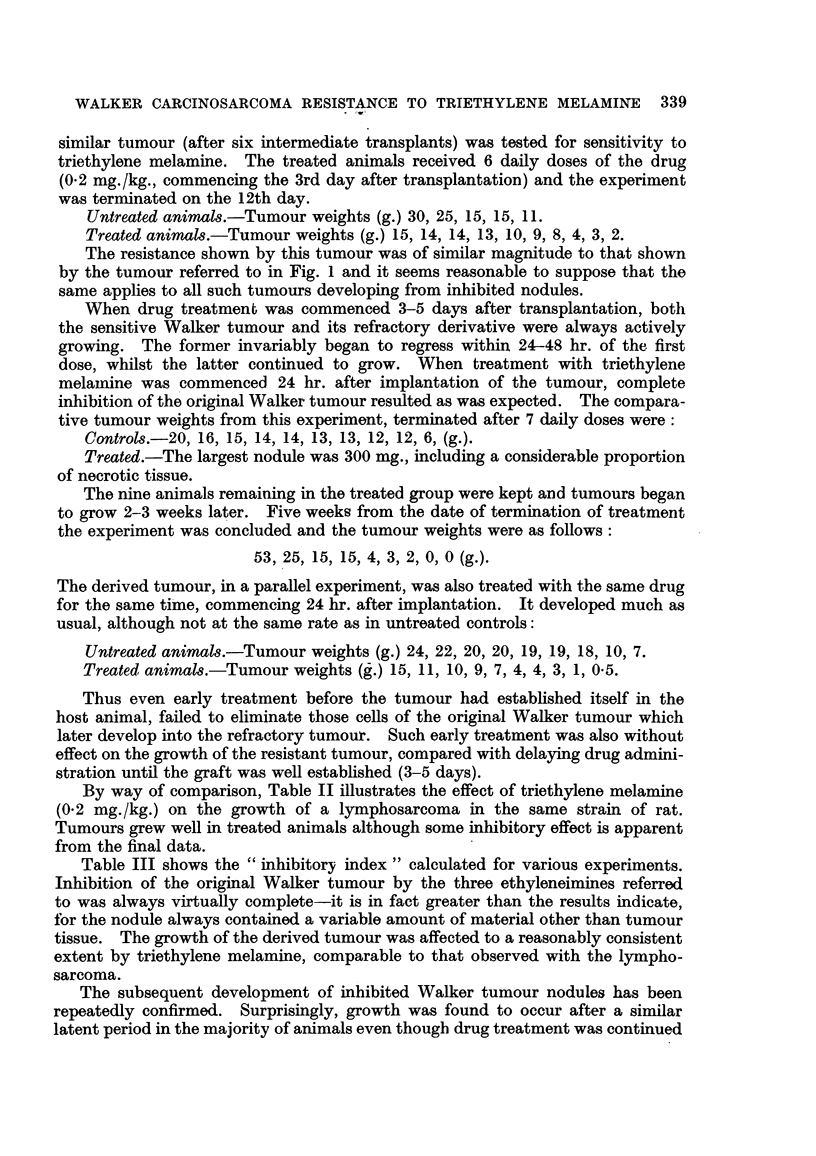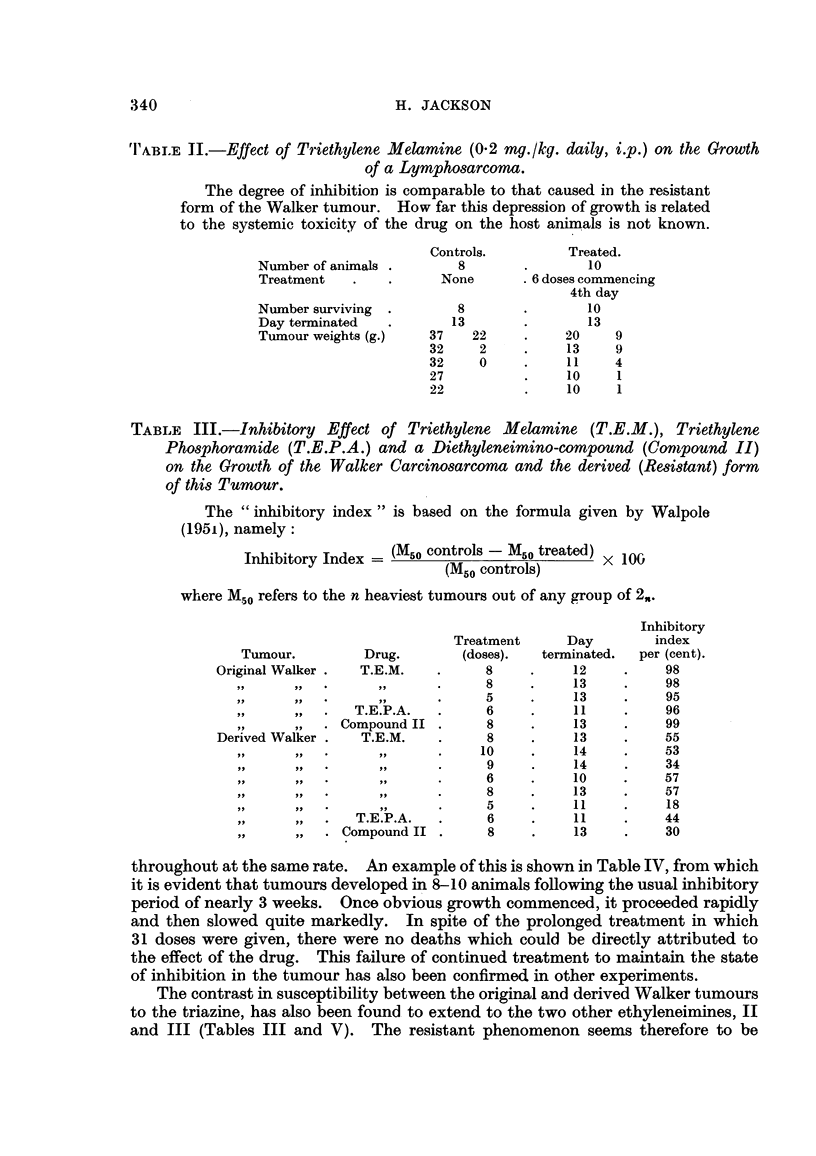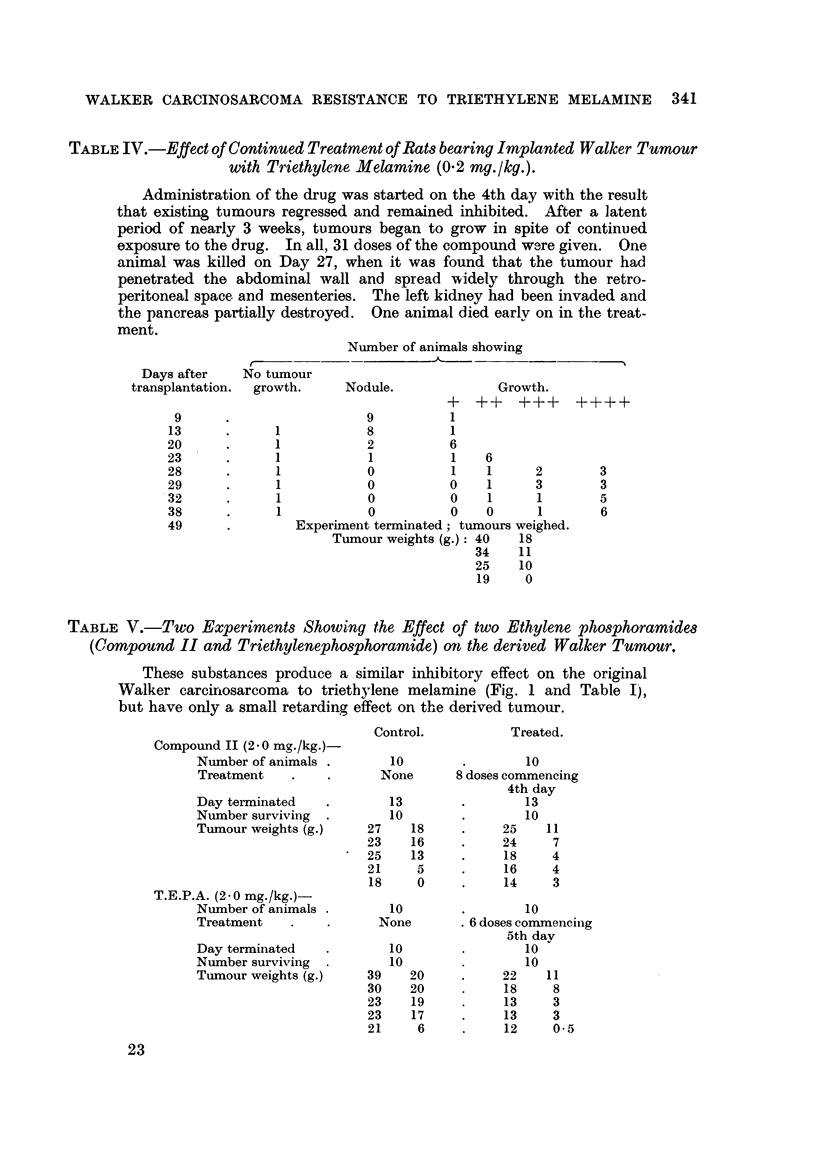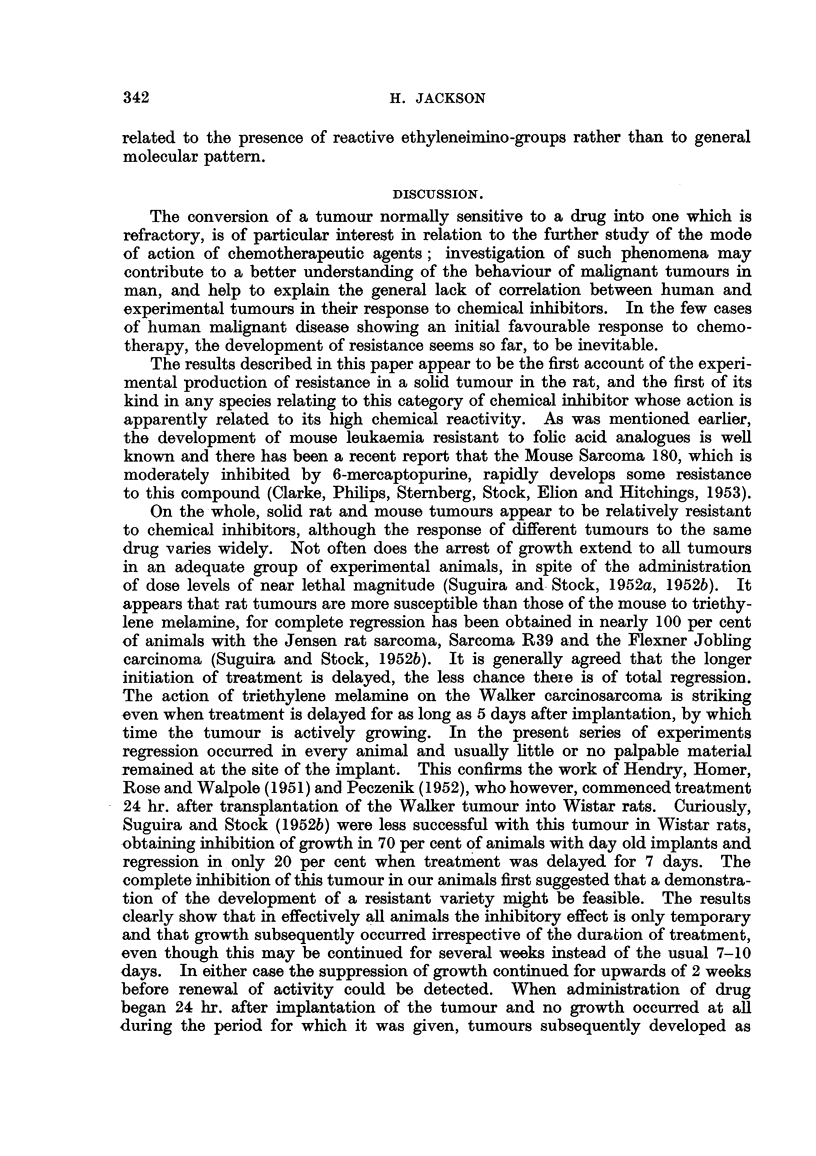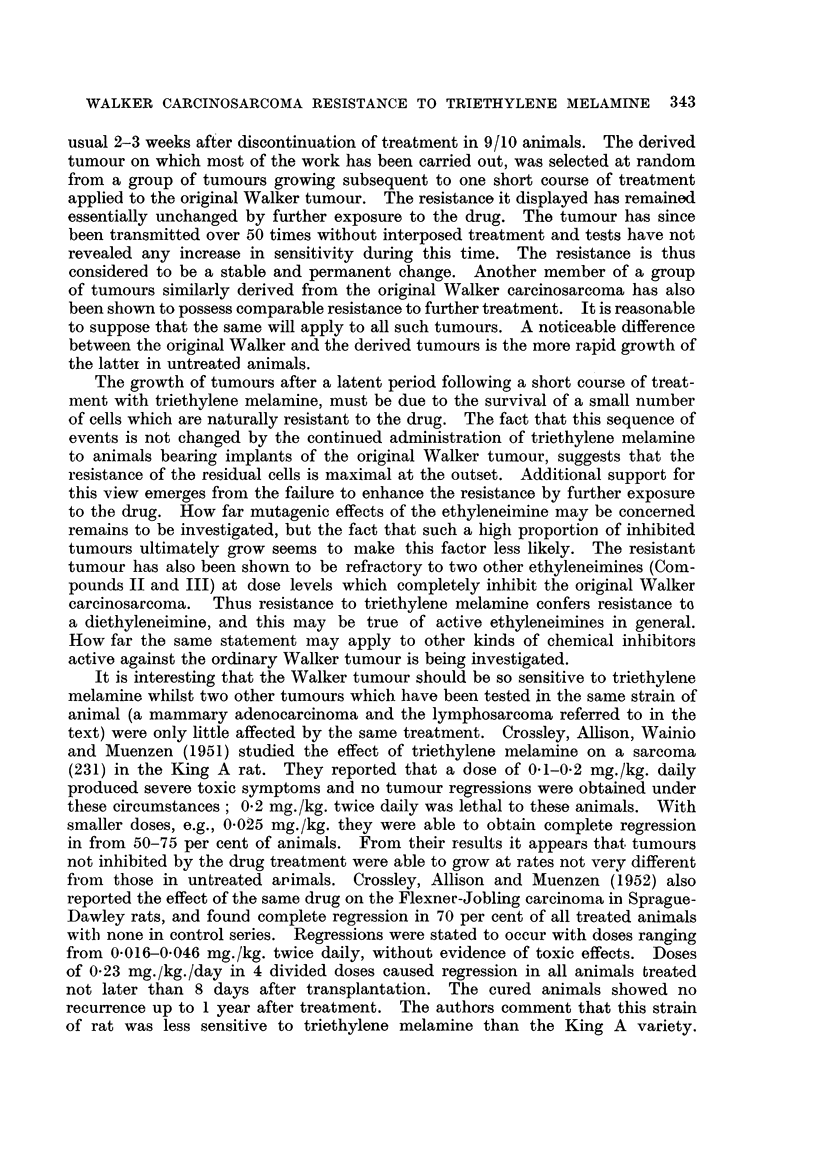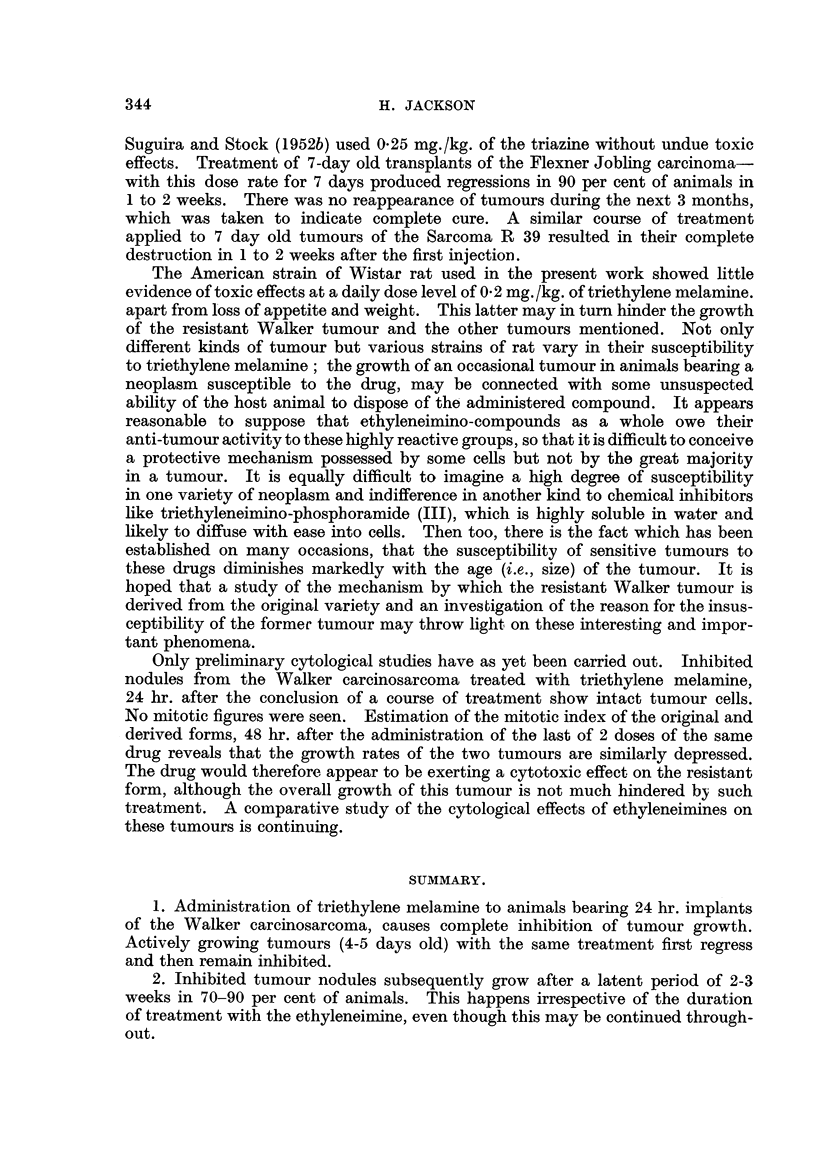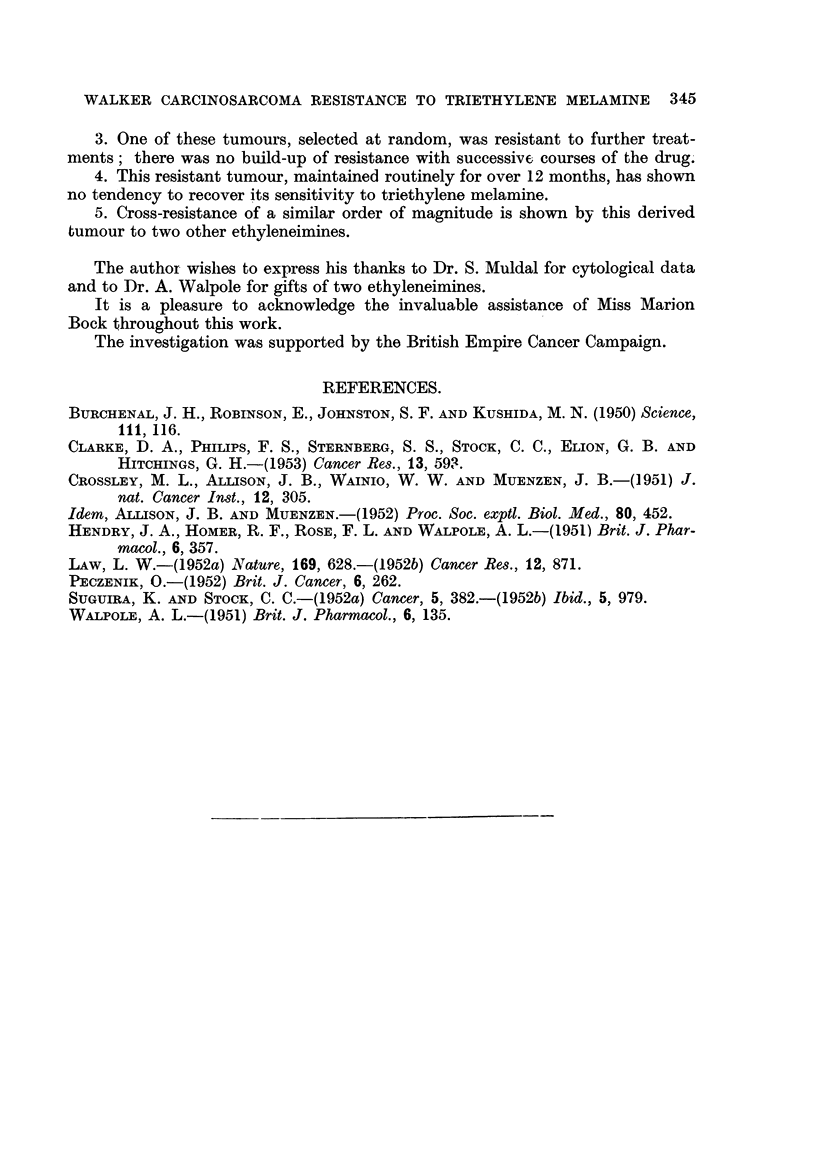# The Development of Resistance in the Walker Carcinosarcoma to the Action of Triethylene Melamine

**DOI:** 10.1038/bjc.1954.34

**Published:** 1954-06

**Authors:** H. Jackson


### THE DEVELOPMENT OF RESISTANCE IN THE WALKER CARCINOSARCOMA TO THE ACTION OF TRIETHYLENE MELAMINE.

H. JACKSON.

*From the Christie Hospital and Holt Radium Institute, Manchester*, 20.

Received for publication February 24, 1954.

THE development of resistance to chemotherapeutic agents is a familiar phenomenon in the investigation and treatment of most diseases of infective origin. It is probable that the production of a refractory state is, as yet, an invariable result in the chemical treatment of those few cases of malignant disease which respond satisfactorily in the first instance.

The appearance of resistance to specific chemical inhibitors has been recognised and investigated in some kinds of mouse leukaemia (Burchenal, Robinson, Johnston and Kushida, 1950; Law, 1952*a*, 1952*b*) but so far it appears that no convincing demonstration of this important phenomenon has been made in solid animal tumours.

This communication records the development of a persistent refractory state in the Walker carcinosarcoma after treatment with triethylene melamine (I), to which substance the tumour is noimally very sensitive; the resistant state is also shown to extend to two related substances, II and III.

I.
2 : 4 : 6-Tris-ethyleneimino-1 : 3 : 5-triazine
(Triethylene melamine)
"T.E.M."

II.

III.

$N^1$, $N^2$, $N^3$-tris-ethylenephosphoramide

(T.E.P.A.).

### MATERIALS AND METHODS.

*Animals.*—Rats of an American Wistar strain werc used, maintained on a cube diet supplied by the North Eastern Agriculture Society. During experiments each animal was allowed 15 g. daily.

*Tumour.*—The Walker carcinosarcoma was originally obtained from the Biological Laboratories of Imperial Chemical Industries. It was easily adapted to these animals and was maintained by subcutaneous dorsal transplantation into young adult rats (110-140 g.).

*Assessment of chemotherapeutic potency.*—Usually groups of ten animals (equal numbers of each sex) were used as controls and similar groups were given daily doses of the drug by the intraperitoneal route, with a double dose on Saturday. Except where stated, dosage was commenced 4–5 days after transplantation. The drug was dissolved in water and kept in the refrigerator, the solution being replaced every few days. Daily inspection of the tumour site was made and the subsequent growth of the neoplasm estimated by palpation using an arbitrary scale of measurement of its size in relation to the bulk of the animal. The animals were killed 10–14 days after implantation and the tumours dissected out and weighed. The decision to terminate an experiment was governed by the rate of growth of the tumour in the control series of animals, which varied somewhat in different experiments. The effect of treatment was measured by comparing the mean tumour weights in control and treated groups and use was also made of the formula suggested by Walpole (1951), which makes allowance for those animals offering undue resistance to the growth of the tumour.

### RESULTS.

Fig. 1 represents the collected results of the main series of experiments. In the first of these, three groups of ten animals were transplanted with the Walker tumour. Ten were used as controls, ten received triethylene melamine, 0·1 mg./kg. daily, and the remainder received twice this dose. Table I shows the result of this experiment on the group receiving the lower dose level; the treated tumours were completely inhibited. The ten animals receiving the higher dose level were not killed, but drug treatment was discontinued after eight consecutive daily doses. Small nodules persisted in the majority of these animals without change for about 2 weeks, after which tumours began to grow; they were well developed in 70 per cent of the group 1 month after cessation of treatment. One

TABLE I.—*Inhibitory Effect of Triethylenemelamine* (0·1 *mg./kg. daily, i.p.*) *on the Walker Carcinosarcoma.*

| | Controls. | | Treated. | |
|---|---|---|---|---|
| Number of animals | 10 | | 10 | |
| Treatment | None | | 8 doses commencing 2nd day | |
| Day terminated | 12 | | 12 | |
| Number surviving | 10 | | 10 | |
| Tumour weights (g.) | 17 | 8 | 0·5 | 0·2 |
| | 12 | 4 | 0·5 | 0·2 |
| | 9 | 0 | 0·4 | 0·1 |
| | 9 | 0 | 0·3 | 0·0 |
| | 9 | 0 | 0·2 | 0·0 |

of the tumours was selected at random, transplanted as before, and subjected to a second course of treatment (Fig. 1). This time, growth occurred in spite of administration of the drug and after 13 days the experiment was terminated. A subsidiary transplant was made from one of the *treated* tumours and a group of ten animals carrying the neoplasm again exposed to the action of the compound. The development of tumours was not markedly depressed compared with the control series and in the next experiment one of these *treated* tumours was once more subjected to a repetition of the above procedure (Fig. 1, fourth treatment). From this time (Fig. 1, " 32 weeks ") one of these tumours (the *derived* tumour)

has been maintained routinely by transplantation at intervals of about 2 weeks—in all about 50 such transferences have since been carried out. From time to time the sensitivity of the tumour to triethylene melamine was tested (Fig. 1) The continued refractory behaviour of the derived tumour compared with the original Walker tumour is clearly evident. This resistance to further treatment shown by such tumours has been confirmed. Thus in another experiment a

FIG. 1.—A representation of the development of the resistant variety from the original sensitive Walker carcinosarcoma, following treatment with triethylene melamin (T.E.M.). The black squares are drawn to scale and represent the mean weight of tumours at the end of an experiment. Experiment 1 shows the effect of the drug (0·1 mg./kg.) on the Walker tumour. The inhibited tumours later grow, and Experiments 2, 3, 4 and 5 show that repeated exposure to the drug has little effect on the growth of the derived tumour ; also that there appears to be no increase in resistance with successive treatments. After Experiment 5, the derived tumour was maintained by transplantation, with occasional tests for susceptibility to the ethyleneimine (Experiments 6A and 7). Experiment 6B represents the result of a test using the diethylenephosphoramide (II) referred to in the text. The control series in the left hand column illustrate the continued susceptibility of the original Walker tumour. In between the experiments referred to above, the resistant tumour was maintained by transplantation about every 10th day.

similar tumour (after six intermediate transplants) was tested for sensitivity to triethylene melamine. The treated animals received 6 daily doses of the drug (0·2 mg./kg., commencing the 3rd day after transplantation) and the experiment was terminated on the 12th day.

*Untreated animals.*—Tumour weights (g.) 30, 25, 15, 15, 11.

*Treated animals.*—Tumour weights (g.) 15, 14, 14, 13, 10, 9, 8, 4, 3, 2.

The resistance shown by this tumour was of similar magnitude to that shown by the tumour referred to in Fig. 1 and it seems reasonable to suppose that the same applies to all such tumours developing from inhibited nodules.

When drug treatment was commenced 3–5 days after transplantation, both the sensitive Walker tumour and its refractory derivative were always actively growing. The former invariably began to regress within 24–48 hr. of the first dose, whilst the latter continued to grow. When treatment with triethylene melamine was commenced 24 hr. after implantation of the tumour, complete inhibition of the original Walker tumour resulted as was expected. The comparative tumour weights from this experiment, terminated after 7 daily doses were :

*Controls.*—20, 16, 15, 14, 14, 13, 13, 12, 12, 6, (g.).

*Treated.*—The largest nodule was 300 mg., including a considerable proportion of necrotic tissue.

The nine animals remaining in the treated group were kept and tumours began to grow 2–3 weeks later. Five weeks from the date of termination of treatment the experiment was concluded and the tumour weights were as follows :

53, 25, 15, 15, 4, 3, 2, 0, 0 (g.).

The derived tumour, in a parallel experiment, was also treated with the same drug for the same time, commencing 24 hr. after implantation. It developed much as usual, although not at the same rate as in untreated controls:

*Untreated animals.*—Tumour weights (g.) 24, 22, 20, 20, 19, 19, 18, 10, 7.

*Treated animals.*—Tumour weights (g.) 15, 11, 10, 9, 7, 4, 4, 3, 1, 0·5.

Thus even early treatment before the tumour had established itself in the host animal, failed to eliminate those cells of the original Walker tumour which later develop into the refractory tumour. Such early treatment was also without effect on the growth of the resistant tumour, compared with delaying drug administration until the graft was well established (3–5 days).

By way of comparison, Table II illustrates the effect of triethylene melamine (0·2 mg./kg.) on the growth of a lymphosarcoma in the same strain of rat. Tumours grew well in treated animals although some inhibitory effect is apparent from the final data.

Table III shows the " inhibitory index " calculated for various experiments. Inhibition of the original Walker tumour by the three ethyleneimines referred to was always virtually complete—it is in fact greater than the results indicate, for the nodule always contained a variable amount of material other than tumour tissue. The growth of the derived tumour was affected to a reasonably consistent extent by triethylene melamine, comparable to that observed with the lymphosarcoma.

The subsequent development of inhibited Walker tumour nodules has been repeatedly confirmed. Surprisingly, growth was found to occur after a similar latent period in the majority of animals even though drug treatment was continued

TABLE II.—*Effect of Triethylene Melamine (0·2 mg./kg. daily, i.p.) on the Growth of a Lymphosarcoma.*

The degree of inhibition is comparable to that caused in the resistant form of the Walker tumour. How far this depression of growth is related to the systemic toxicity of the drug on the host animals is not known.

| | Controls. | | Treated. | |
|---|---|---|---|---|
| Number of animals | 8 | | 10 | |
| Treatment | None | | 6 doses commencing 4th day | |
| Number surviving | 8 | | 10 | |
| Day terminated | 13 | | 13 | |
| Tumour weights (g.) | 37 | 22 | 20 | 9 |
| | 32 | 2 | 13 | 9 |
| | 32 | 0 | 11 | 4 |
| | 27 | | 10 | 1 |
| | 22 | | 10 | 1 |

TABLE III.—*Inhibitory Effect of Triethylene Melamine (T.E.M.), Triethylene Phosphoramide (T.E.P.A.) and a Diethyleneimino-compound (Compound II) on the Growth of the Walker Carcinosarcoma and the derived (Resistant) form of this Tumour.*

The "inhibitory index" is based on the formula given by Walpole (1951), namely:

$$\text{Inhibitory Index} = \frac{(M_{50}\text{ controls} - M_{50}\text{ treated})}{(M_{50}\text{ controls})} \times 100$$

where $M_{50}$ refers to the $n$ heaviest tumours out of any group of $2_n$.

| Tumour. | Drug. | Treatment (doses). | Day terminated. | Inhibitory index per (cent). |
|---|---|---|---|---|
| Original Walker | T.E.M. | 8 | 12 | 98 |
| ,, ,, | ,, | 8 | 13 | 98 |
| ,, ,, | ,, | 5 | 13 | 95 |
| ,, ,, | T.E.P.A. | 6 | 11 | 96 |
| ,, ,, | Compound II | 8 | 13 | 99 |
| Derived Walker | T.E.M. | 8 | 13 | 55 |
| ,, ,, | ,, | 10 | 14 | 53 |
| ,, ,, | ,, | 9 | 14 | 34 |
| ,, ,, | ,, | 6 | 10 | 57 |
| ,, ,, | ,, | 8 | 13 | 57 |
| ,, ,, | ,, | 5 | 11 | 18 |
| ,, ,, | T.E.P.A. | 6 | 11 | 44 |
| ,, ,, | Compound II | 8 | 13 | 30 |

throughout at the same rate. An example of this is shown in Table IV, from which it is evident that tumours developed in 8–10 animals following the usual inhibitory period of nearly 3 weeks. Once obvious growth commenced, it proceeded rapidly and then slowed quite markedly. In spite of the prolonged treatment in which 31 doses were given, there were no deaths which could be directly attributed to the effect of the drug. This failure of continued treatment to maintain the state of inhibition in the tumour has also been confirmed in other experiments.

The contrast in susceptibility between the original and derived Walker tumours to the triazine, has also been found to extend to the two other ethyleneimines, II and III (Tables III and V). The resistant phenomenon seems therefore to be

TABLE IV.—*Effect of Continued Treatment of Rats bearing Implanted Walker Tumour with Triethylene Melamine* (0·2 *mg./kg.*).

Administration of the drug was started on the 4th day with the result that existing tumours regressed and remained inhibited. After a latent period of nearly 3 weeks, tumours began to grow in spite of continued exposure to the drug. In all, 31 doses of the compound were given. One animal was killed on Day 27, when it was found that the tumour had penetrated the abdominal wall and spread widely through the retroperitoneal space and mesenteries. The left kidney had been invaded and the pancreas partially destroyed. One animal died early on in the treatment.

| | Number of animals showing | | | | | |
|---|---|---|---|---|---|---|
| Days after transplantation. | No tumour growth. | Nodule. | Growth. | | | |
| | | | + | ++ | +++ | ++++ |
| 9 | | 9 | 1 | | | |
| 13 | 1 | 8 | 1 | | | |
| 20 | 1 | 2 | 6 | | | |
| 23 | 1 | 1 | 1 | 6 | | |
| 28 | 1 | 0 | 1 | 1 | 2 | 3 |
| 29 | 1 | 0 | 0 | 1 | 3 | 3 |
| 32 | 1 | 0 | 0 | 1 | 1 | 5 |
| 38 | 1 | 0 | 0 | 0 | 1 | 6 |
| 49 | Experiment terminated ; tumours weighed. | | | | | |
| | | Tumour weights (g.) : | | 40 | 18 | |
| | | | | 34 | 11 | |
| | | | | 25 | 10 | |
| | | | | 19 | 0 | |

TABLE V.—*Two Experiments Showing the Effect of two Ethylene phosphoramides (Compound II and Triethylenephosphoramide) on the derived Walker Tumour.*

These substances produce a similar inhibitory effect on the original Walker carcinosarcoma to triethylene melamine (Fig. 1 and Table I), but have only a small retarding effect on the derived tumour.

| | Control. | | Treated. | |
|---|---|---|---|---|
| Compound II (2·0 mg./kg.)— | | | | |
| Number of animals | 10 | | 10 | |
| Treatment | None | | 8 doses commencing 4th day | |
| Day terminated | 13 | | 13 | |
| Number surviving | 10 | | 10 | |
| Tumour weights (g.) | 27 | 18 | 25 | 11 |
| | 23 | 16 | 24 | 7 |
| | 25 | 13 | 18 | 4 |
| | 21 | 5 | 16 | 4 |
| | 18 | 0 | 14 | 3 |
| T.E.P.A. (2·0 mg./kg.)— | | | | |
| Number of animals | 10 | | 10 | |
| Treatment | None | | 6 doses commencing 5th day | |
| Day terminated | 10 | | 10 | |
| Number surviving | 10 | | 10 | |
| Tumour weights (g.) | 39 | 20 | 22 | 11 |
| | 30 | 20 | 18 | 8 |
| | 23 | 19 | 13 | 3 |
| | 23 | 17 | 13 | 3 |
| | 21 | 6 | 12 | 0·5 |

related to the presence of reactive ethyleneimino-groups rather than to general molecular pattern.

### DISCUSSION.

The conversion of a tumour normally sensitive to a drug into one which is refractory, is of particular interest in relation to the further study of the mode of action of chemotherapeutic agents; investigation of such phenomena may contribute to a better understanding of the behaviour of malignant tumours in man, and help to explain the general lack of correlation between human and experimental tumours in their response to chemical inhibitors. In the few cases of human malignant disease showing an initial favourable response to chemotherapy, the development of resistance seems so far, to be inevitable.

The results described in this paper appear to be the first account of the experimental production of resistance in a solid tumour in the rat, and the first of its kind in any species relating to this category of chemical inhibitor whose action is apparently related to its high chemical reactivity. As was mentioned earlier, the development of mouse leukaemia resistant to folic acid analogues is well known and there has been a recent report that the Mouse Sarcoma 180, which is moderately inhibited by 6-mercaptopurine, rapidly develops some resistance to this compound (Clarke, Philips, Sternberg, Stock, Elion and Hitchings, 1953).

On the whole, solid rat and mouse tumours appear to be relatively resistant to chemical inhibitors, although the response of different tumours to the same drug varies widely. Not often does the arrest of growth extend to all tumours in an adequate group of experimental animals, in spite of the administration of dose levels of near lethal magnitude (Suguira and Stock, 1952*a*, 1952*b*). It appears that rat tumours are more susceptible than those of the mouse to triethylene melamine, for complete regression has been obtained in nearly 100 per cent of animals with the Jensen rat sarcoma, Sarcoma R39 and the Flexner Jobling carcinoma (Suguira and Stock, 1952*b*). It is generally agreed that the longer initiation of treatment is delayed, the less chance there is of total regression. The action of triethylene melamine on the Walker carcinosarcoma is striking even when treatment is delayed for as long as 5 days after implantation, by which time the tumour is actively growing. In the present series of experiments regression occurred in every animal and usually little or no palpable material remained at the site of the implant. This confirms the work of Hendry, Homer, Rose and Walpole (1951) and Peczenik (1952), who however, commenced treatment 24 hr. after transplantation of the Walker tumour into Wistar rats. Curiously, Suguira and Stock (1952*b*) were less successful with this tumour in Wistar rats, obtaining inhibition of growth in 70 per cent of animals with day old implants and regression in only 20 per cent when treatment was delayed for 7 days. The complete inhibition of this tumour in our animals first suggested that a demonstration of the development of a resistant variety might be feasible. The results clearly show that in effectively all animals the inhibitory effect is only temporary and that growth subsequently occurred irrespective of the duration of treatment, even though this may be continued for several weeks instead of the usual 7–10 days. In either case the suppression of growth continued for upwards of 2 weeks before renewal of activity could be detected. When administration of drug began 24 hr. after implantation of the tumour and no growth occurred at all during the period for which it was given, tumours subsequently developed as usual 2–3 weeks after discontinuation of treatment in 9/10 animals. The derived tumour on which most of the work has been carried out, was selected at random from a group of tumours growing subsequent to one short course of treatment applied to the original Walker tumour. The resistance it displayed has remained essentially unchanged by further exposure to the drug. The tumour has since been transmitted over 50 times without interposed treatment and tests have not revealed any increase in sensitivity during this time. The resistance is thus considered to be a stable and permanent change. Another member of a group of tumours similarly derived from the original Walker carcinosarcoma has also been shown to possess comparable resistance to further treatment. It is reasonable to suppose that the same will apply to all such tumours. A noticeable difference between the original Walker and the derived tumours is the more rapid growth of the latter in untreated animals.

The growth of tumours after a latent period following a short course of treatment with triethylene melamine, must be due to the survival of a small number of cells which are naturally resistant to the drug. The fact that this sequence of events is not changed by the continued administration of triethylene melamine to animals bearing implants of the original Walker tumour, suggests that the resistance of the residual cells is maximal at the outset. Additional support for this view emerges from the failure to enhance the resistance by further exposure to the drug. How far mutagenic effects of the ethyleneimine may be concerned remains to be investigated, but the fact that such a high proportion of inhibited tumours ultimately grow seems to make this factor less likely. The resistant tumour has also been shown to be refractory to two other ethyleneimines (Compounds II and III) at dose levels which completely inhibit the original Walker carcinosarcoma. Thus resistance to triethylene melamine confers resistance to a diethyleneimine, and this may be true of active ethyleneimines in general. How far the same statement may apply to other kinds of chemical inhibitors active against the ordinary Walker tumour is being investigated.

It is interesting that the Walker tumour should be so sensitive to triethylene melamine whilst two other tumours which have been tested in the same strain of animal (a mammary adenocarcinoma and the lymphosarcoma referred to in the text) were only little affected by the same treatment. Crossley, Allison, Wainio and Muenzen (1951) studied the effect of triethylene melamine on a sarcoma (231) in the King A rat. They reported that a dose of 0·1–0·2 mg./kg. daily produced severe toxic symptoms and no tumour regressions were obtained under these circumstances ; 0·2 mg./kg. twice daily was lethal to these animals. With smaller doses, e.g., 0·025 mg./kg. they were able to obtain complete regression in from 50–75 per cent of animals. From their results it appears that tumours not inhibited by the drug treatment were able to grow at rates not very different from those in untreated animals. Crossley, Allison and Muenzen (1952) also reported the effect of the same drug on the Flexner-Jobling carcinoma in Sprague-Dawley rats, and found complete regression in 70 per cent of all treated animals with none in control series. Regressions were stated to occur with doses ranging from 0·016–0·046 mg./kg. twice daily, without evidence of toxic effects. Doses of 0·23 mg./kg./day in 4 divided doses caused regression in all animals treated not later than 8 days after transplantation. The cured animals showed no recurrence up to 1 year after treatment. The authors comment that this strain of rat was less sensitive to triethylene melamine than the King A variety.

Suguira and Stock (1952*b*) used 0·25 mg./kg. of the triazine without undue toxic effects. Treatment of 7-day old transplants of the Flexner Jobling carcinoma—with this dose rate for 7 days produced regressions in 90 per cent of animals in 1 to 2 weeks. There was no reappearance of tumours during the next 3 months, which was taken to indicate complete cure. A similar course of treatment applied to 7 day old tumours of the Sarcoma R 39 resulted in their complete destruction in 1 to 2 weeks after the first injection.

The American strain of Wistar rat used in the present work showed little evidence of toxic effects at a daily dose level of 0·2 mg./kg. of triethylene melamine. apart from loss of appetite and weight. This latter may in turn hinder the growth of the resistant Walker tumour and the other tumours mentioned. Not only different kinds of tumour but various strains of rat vary in their susceptibility to triethylene melamine ; the growth of an occasional tumour in animals bearing a neoplasm susceptible to the drug, may be connected with some unsuspected ability of the host animal to dispose of the administered compound. It appears reasonable to suppose that ethyleneimino-compounds as a whole owe their anti-tumour activity to these highly reactive groups, so that it is difficult to conceive a protective mechanism possessed by some cells but not by the great majority in a tumour. It is equally difficult to imagine a high degree of susceptibility in one variety of neoplasm and indifference in another kind to chemical inhibitors like triethyleneimino-phosphoramide (III), which is highly soluble in water and likely to diffuse with ease into cells. Then too, there is the fact which has been established on many occasions, that the susceptibility of sensitive tumours to these drugs diminishes markedly with the age (*i.e.*, size) of the tumour. It is hoped that a study of the mechanism by which the resistant Walker tumour is derived from the original variety and an investigation of the reason for the insusceptibility of the former tumour may throw light on these interesting and important phenomena.

Only preliminary cytological studies have as yet been carried out. Inhibited nodules from the Walker carcinosarcoma treated with triethylene melamine, 24 hr. after the conclusion of a course of treatment show intact tumour cells. No mitotic figures were seen. Estimation of the mitotic index of the original and derived forms, 48 hr. after the administration of the last of 2 doses of the same drug reveals that the growth rates of the two tumours are similarly depressed. The drug would therefore appear to be exerting a cytotoxic effect on the resistant form, although the overall growth of this tumour is not much hindered by such treatment. A comparative study of the cytological effects of ethyleneimines on these tumours is continuing.

### SUMMARY.

1. Administration of triethylene melamine to animals bearing 24 hr. implants of the Walker carcinosarcoma, causes complete inhibition of tumour growth. Actively growing tumours (4-5 days old) with the same treatment first regress and then remain inhibited.

2. Inhibited tumour nodules subsequently grow after a latent period of 2-3 weeks in 70–90 per cent of animals. This happens irrespective of the duration of treatment with the ethyleneimine, even though this may be continued throughout.

3. One of these tumours, selected at random, was resistant to further treatments; there was no build-up of resistance with successive courses of the drug.

4. This resistant tumour, maintained routinely for over 12 months, has shown no tendency to recover its sensitivity to triethylene melamine.

5. Cross-resistance of a similar order of magnitude is shown by this derived tumour to two other ethyleneimines.

The author wishes to express his thanks to Dr. S. Muldal for cytological data and to Dr. A. Walpole for gifts of two ethyleneimines.

It is a pleasure to acknowledge the invaluable assistance of Miss Marion Bock throughout this work.

The investigation was supported by the British Empire Cancer Campaign.

### REFERENCES.

Burchenal, J. H., Robinson, E., Johnston, S. F. and Kushida, M. N. (1950) *Science*, **111**, 116.

Clarke, D. A., Philips, F. S., Sternberg, S. S., Stock, C. C., Elion, G. B. and Hitchings, G. H.—(1953) *Cancer Res.*, **13**, 593.

Crossley, M. L., Allison, J. B., Wainio, W. W. and Muenzen, J. B.—(1951) *J. nat. Cancer Inst.*, **12**, 305.

*Idem*, Allison, J. B. and Muenzen.—(1952) *Proc. Soc. exptl. Biol. Med.*, **80**, 452.

Hendry, J. A., Homer, R. F., Rose, F. L. and Walpole, A. L.—(1951) *Brit. J. Pharmacol.*, **6**, 357.

Law, L. W.—(1952*a*) *Nature*, **169**, 628.—(1952*b*) *Cancer Res.*, **12**, 871.

Peczenik, O.—(1952) *Brit. J. Cancer*, **6**, 262.

Suguira, K. and Stock, C. C.—(1952*a*) *Cancer*, **5**, 382.—(1952*b*) *Ibid.*, **5**, 979.

Walpole, A. L.—(1951) *Brit. J. Pharmacol.*, **6**, 135.